# Utilizing Dichloromethane as an Extremely Proficient Substitute for Phenol/Chloroform in Extracting RNA with Exceptional Purity from Woody Tissues of Coconut

**DOI:** 10.3390/mps6050075

**Published:** 2023-08-26

**Authors:** Amjad Iqbal, Yaodong Yang

**Affiliations:** 1 Hainan Key Laboratory of Tropical Oil Crops Biology/Coconut Research Institute, Chinese Academy of Tropical Agricultural Science, Wenchang 571339, China; 2 Department of Food Science & Technology, Abdul Wali Khan University, Mardan 23200, Pakistan

**Keywords:** dichloromethane, RNA extraction, cDNA library, RT-PCR, TRIZOL, CTAB

## Abstract

Procuring high-grade RNA from mature coconut tissues is a tricky and labor-intensive process due to the intricate scaffold of polysaccharides, polyphenols, lipids, and proteins that form firm complexes with nucleic acids. However, we have effectively developed a novel method for the first time, letting the retrieval of high-grade RNA from the roots, endosperm, and mesocarp of mature coconut trees take place. In this method, we exploited dichloromethane as a replacement to phenol/chloroform for RNA recovery from mature coconut tissues. The amount of high-grade RNA acquired from the roots of mature coconut trees was 120.7 µg/g, with an A260/280 ratio of 1.95. Similarly, the mature coconut mesocarp yielded 134.6 µg/g FW of quality RNA with A260/280 ratio of 1.98, whereas the mature coconut endosperm produced 120.4 µg/g FW of quality RNA with A260/280 ratio of 2.01. Furthermore, the RNA isolation using the dichloromethane method exhibited excellent performance in downstream experiments, particularly in RT-PCR for cDNA production and amplification. On the contrary, the RNA plant kit, TRIZOL, and Cetyl Trimethyl Ammonium Bromide (CTAB) methods were unsuccessful in isolating substantial quantities of RNA with exceptional purities from the mentioned coconut tissues. In view of these findings, we conclude that the newly developed method will be pivotal in effectively extracting RNA with high purity from mature coconut tissues.

## 1. Introduction

Plant tissues predominantly comprise polysaccharides, proteins, lipids, and polyphenols, which have a tendency to bind with RNA and disrupt its segregation process. These macro-biomolecules precipitate alongside RNA, leading to significant impacts on the integrity and amount of the isolated RNA [1,2,3]. It is also conceivable that the polyphenols present in the lignified tissues of mature plants can bind to the RNA, impeding its segregation process [4,5]. Indeed, for successful downstream experiments like constructing a complementary DNA (cDNA) library, real-time PCR, reverse transcription, RNA sequencing, and others, it is essential to isolate unmodified RNA with exceptional purity [6,7,8,9]. To achieve this objective, researchers have employed various methods to acquire superior quality nucleic acids from complex plant tissues [10,11,12,13]. For the procurement of superior quality RNA from carbohydrate or lipid-dense plant tissues of *Brassica napus* [14], *Arabidopsis thaliana* [15], and *Oryza sativa* [16], commercial kits developed by Invitrogen TRIZOL reagents are widely accessible in the market. The Tiangen RNA Plant Reagent Kit, developed by Tiangen company, is specifically designed for the segregation of RNA with exceptional purity in larger quantities from starch and polyphenol-dense tissues of apple, banana, pear, and potato [17]. Despite the success of the Tiangen RNA Plant Reagent Kit and Invitrogen TRIZOL reagents in extracting quality RNA from certain plant tissues, both methods have been ineffective in procuring RNA with superior integrity from the Palmaceae family. The tissues of this family are abundant in phenolic, polysaccharides, and fat, which present significant challenges for RNA extraction [7]. Likewise, CTAB (Cetyl Trimethyl Ammonium Bromide) is one of the extensively adopted techniques for the segregation of RNA with exceptional purity from intricate plant matrices [2]. Nevertheless, the main disadvantage of using CTAB is the extended isolation process, which can potentially lead to RNA degradation.

In order to overcome these challenges, the Coconut Research Institute (CRI) has undertaken significant endeavors to isolate substantial quantities of premium-quality RNA from young coconut tissues. These tissues are rich in primary cell wall components, lipids, and other complex biomolecules. To date, the CRI has successfully established methods for isolating high-quality RNA from diverse sources, including palm leaves [7], coconut endosperm, mesocarp, apple buds, and leaves [10,12,13]. Although the methods yielded consistent results for acquiring pure RNA from young coconut tissues, they proved ineffective in extracting quality RNA from mature coconut tree roots and mature coconut mesocarp, which contain high levels of lignin/secondary cell walls. To address this limitation, a new method based on dichloromethane extraction has been developed. This novel approach is reliable, cost-effective, and rapid in isolating appreciable quantities of pure RNA from the hard woody tissues of coconut. Initially designed for RNA extraction from the woody tissues of coconut species, the dichloromethane-based method can also be applied to get pure RNA from other plant and animal tissues, offering broader applicability.

## 2. Experimental Design

Triplicated samples of mature coconut tree roots and mesocarp were collected to ensure robust statistical analysis. The study employed four different RNA extraction methods. The dichloromethane method served as the primary experimental group for extracting RNA from mature coconut tree roots and mesocarp. Additionally, the RNA plant kit was used following the manufacturer’s instructions, the TRIZOL reagent was used according to the standard protocol, and the CTAB method was applied as previously described by Wang and Stegemann [2] to extract RNA from the said tissues for comparison.

### 2.1. Materials

Numerous laboratory reagents and equipment were used in the study, including agarose gel, isopropanol, sodium acetate, guanidine thiocynate, β-merceptoethanol, glycerol, Polyvinylpyrrolidone-40 (PVP-40), pipette, mortar and pestle, Ribonuclease-free tubes, plastic bags, spatula, cutting tools, freezer, DPEC water, ethidium bromide, Tris borate EDTA (TBE) buffer, and DNA ladder. The reagents employed in this study were of superior quality and acquired from Chemical Reagents and Real Times, China. For reverse transcription, a TaKaRa PrimeScript^TM^ II 1st strand cDNA synthesis kit was utilized, and a Real Time PCR kit was employed for amplifying the cDNA in the PCR process. 


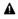
 *Cautions!*

Ethidium bromide is a perilous chemical and must be managed with utmost caution in a fume hood. The other reagents used may also have certain effects, so it is important to avoid eye and skin contact while working with them. Safety precautions should be taken when handling all chemicals to ensure a safe laboratory environment.

Prior to the experiment, all glassware, spatulas, and mortars and pestles must be disinfected at 121 °C for 20 min. After autoclaving, they must be in folded in aluminum foil and be kept in a hot air oven until the time of use to prevent contamination from extrinsic RNase. Taking these precautions ensures a clean and sterile environment for the experiment.

### 2.2. Equipment

- Vortex (Vortex-BE1, Kylin-Bell Lab Instruments, Nantong, China);
- Bench centrifuge (Eppendorf Centrifuge 5804 R);
- PCR machine/thermocycler (TaKaRa thermocycler dice touch TP350, TaKaRa Bio Incharge, Le Pecq, France);
- Nanodrop (Nanodrop 2000 spectrophotometer, Thermo Scientific, Waltham, MA, USA);
- Agrose gel electrophoresis system;
- Gel Doc system (Syngene G: Box F3, Gene Company Limited, Hong Kong, China).

### 2.3. Plant Materials

Root tissues were obtained from a mature coconut tree, whereas mesocarp and endosperm samples were collected from mature coconuts harvested from a tree at the CRI, China. Additionally, samples from various tissues of coconut (apple, coconut leaf bud, and coconut leaf) were collected from newly planted coconuts at CRI. Upon collection, every specimen was delicately chopped into tiny sections using sanitized shears or a sharp blade and moved into zipper bags. The bags containing the samples were finally rapidly immersed in liquid nitrogen and subsequently stored at −80 °C until subsequent examination. 

### 2.4. The RNA Extraction Buffer

To prepare a 100 mL buffer solution, add

- Guanidine thiocyanate             : 23 g
- 3M sodium acetate (pH 5)       : 3 mL
- Glycerol                                     : 4 mL
- ddH_2_O or DEPC water            : up to 100 mL
- PVP-40                                      : 1.6%
- β-marceptoethanol                   : 1.9%

Take DEPC water (60 mL) and sequentially blend the ingredients with it, making sure to reach a total volume of 100 mL through the addition of DEPC water. The resulting buffer should be stored in the refrigerator. 

## 3. Procedure

### 3.1. Tissue Preparation and Maceration

1. Before homogenizing the tissue, the sample-filled zipper bags from the refrigerator were moved to liquid nitrogen to prevent defrosting.

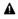
Critical step

Defrosting could lead to RNA degradation by RNase enzymes.

2. The sterile mortar and pestle, previously autoclaved, were cooled down by the addition of liquid nitrogen.
3. Approximately 0.1 g of refrigerated sample was taken from the zipper bag and moved into the liquid nitrogen and PVP-40-holding mortar. The sample was then finely powdered using the pestle, with the mortar being periodically refilled with liquid nitrogen in the course of milling process to avoid defrosting.

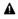
Critical step 

Due to its significance and sensitivity, the homogenization process required careful and meticulous handling:

(a) To elude sample loss, it is essential to add liquid nitrogen slowly to the mortar, taking care to prevent splashing during the process.
(b) Preventing sample defrosting is crucial since it can cause the liberation of RNA from disrupted cells, rendering it susceptible to degradation by RNase enzymes.
(c) To ensure the prevention of sample cross-contamination with RNase, it is vital to carry out this step in a fume hood.

### 3.2. Extraction of RNA

4. Using a frozen spatula, the ground sample was rapidly shifted into an eppendorf tube holding 700 μL of the extraction buffer. After vortexing the contents of the tube for 15 s, we introduced 500 μL of dichloromethane, vortexed it again for 15 s, and, finally, centrifuged the tube for 5 min at 16,639 rcf and 4 °C.

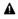
Critical step 

It was crucial to ensure that whole sample was shifted to the Ribonuclease-free tube having the extraction buffer. Neglecting this step may cause substantial loss of RNA.

### 3.3. Precipitation of RNA

5. We gently moved 500 µL of top aqueous fraction of the solution to a new tube, ensuring that the tube was Ribonuclease-free. Subsequently, we introduced the same amount of chilled isopropanol into the tube, inverted the tubes to mix the solution, and set it aside at ambient temperature for 5 min.

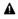
Critical step 

With utmost care, we moved the supernatant to a new tube that was free from RNase to prevent any potential contamination. The utilization of chilled isopropanol expedited the process by rapidly precipitating RNA within a short period of time.

6. After the incubation, we centrifuged the contents of the tube at 16,639 rcf and 4 °C for 5 min and subsequently disposed of the supernatant. The nucleic acids aggregated at the base of the tube.

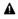
Critical step 

Prior to drying the pellet, it was essential to mark its location on the exterior of the tube. Drying could often render the pellet translucent, which could make it challenging to locate afterward. Furthermore, thorough drying of the pellet could pose challenges when attempting to dissolve it later on.

7. The pellet was rinsed with 1 mL of 75% ethanol, accompanied by centrifugation at 4 °C and 11,000 rcf for 5 min. The washing step was repeated once more in the same manner.
8. The mixture was supplemented with 50 μL of DPEC and 1 μL of DNAse (to collect RNA) and then incubated at 37 °C for 30 min.
9. We added the same amount of chilled isopropanol into the tube, inverted the tubes to mix the solution, and set it aside at ambient temperature for 5 min. After incubation we centrifuged the contents of the tube at 16,639 rcf and 4 °C for 5 min and subsequently disposed of the supernatant. The RNA aggregated at the base of the tube.
10. The pellet was rinsed with 1 mL of 75% ethanol, accompanied by centrifugation at 4 °C and 11,000 rcf for 5 min. The washing step was repeated once more in the same manner.
11. After drying the pellet for 5 min, we carefully re-dissolved the RNA in 20 μL of Ribonuclease-free (DEPC) water using a pipette until it was fully dissolved.

### 3.4. RNA Characterization and Quantification

10. The RNA abundance and integrity were evaluated using the NanoDrop, whereas the purity was validated through agarose gel electrophoresis.

(a) The amount of RNA was assayed spectrophotometrically using NanoDrop 2000 analysis. A sample (1 μL) was loaded onto the NanoDrop 2000, and the optical density was measured at 260 and 280 nm. The quantity of RNA was calculated using the absorbance at A260, whereas the purity of the RNA was assessed based on the A260/280 ratio.
(b) To set up the agarose gel for electrophoresis, agarose (0.2 g) was dissolved in 25 mL of 0.5x TBE buffer in a Ribonuclease-free conical flask, resulting in a 0.8% (*w*/*v*) agarose gel. The conical flask was subjected to microwave heating until the agarose was fully dispersed in TBE buffer. Following that, ethidium bromide (2 μL) was introduced to the contents and stirred to achieve thorough mixing. The mixture was deposited inside the gel tray alongside a comb and allowed to solidify for about 20 min. Once the gel had solidified, RNA-loading buffer (2 μL) was combined with the RNA sample (3 μL), and the resulting mixture was introduced to the gel. The gel was subsequently subjected to electrophoresis at 120 V and 240 mA for 12 min. Once the electrophoresis process was complete, the gel bands were spotted, we recorded the Gel Doc system to capture images, and saved the files. Microsoft Office Picture Manager was utilized for cropping the gel images.

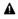
Critical step 

To prevent any potential contamination, it was imperative that all components of the assembly were Ribonuclease-free. Furthermore, it was essential to ensure that the RNA loading buffer and TBE were devoid of RNase contamination. Failure to ensure Ribonuclease-free conditions may result in false negative results, compromising the accuracy of the analysis.

### 3.5. cDNA Generation

The TaKaRa PrimeScriptTM II 1st strand cDNA synthesis kit was employed for first-strand cDNA synthesis. Adhering to the manufacturer’s instructions, the RT reaction was performed according to the following procedure; roughly, the procured RNA (2 μg) was blended with 1 μL of oligo dT primer (50 μM) + dNTP mixture (10 mM), and the quantity was set to 10 μL, employing DPEC water in a Microtube. After incubating the tube at 65 °C for 5 min, it was cooled on ice. Afterward, 4 μL of 5x PrimeScriptTM II buffer, 0.5 μL of ribonuclease inhibitor, and 1 μL of PrimeScriptTM II RTase were incorporated, and the overall volume was brought to 20 μL with DPEC water. The mixture was placed in an incubator at 42 °C for 60 min to facilitate cDNA synthesis. To render the enzymes inactive, the mixture was subjected to an additional incubation of 5 min at 95 °C, and then it was cooled on ice.

### 3.6. RT-PCR 

Subsequent to the production of first-strand cDNA, RT-PCR was conducted exploiting a Real Time kit to augment the cDNA. In coconut tree root and mature mesocarp samples, a housekeeping gene Actin was examined, utilizing the following primers designed by the Biotechnology lab at CRI, CATAS: CnACT-For (ATAAAGTATGGCTGATGCTGAGG) and CnACT-Re (CAACAATGCTTGGGAACACA). The cDNAs were amplified by blending cDNA (2 μL), reverse primer (1 μL of 10 μM), forward primer (1 µL of 10 μM), 10x TAQ buffer (5 μL), dNTP mix (1 μL of 10 mM), TAQ (0.5 µL of 5U/ µL) from TaKaRa, and ddH_2_O (39.5 μL) in a micro-centrifuge tube. Afterward, the tube was moved to a thermocycler with the designated conditions: 94 °C for 3 min; followed by 35 cycles of 94 °C for 30 s, 54 °C for 30 s, and 72 °C for 1 min; and a final extension step at 72 °C for 5 min. To verify the absence of chromosomal DNA contamination, a control experiment was conducted using RNA without the presence of reverse transcriptase.

## 4. Results

**Figure 1:**
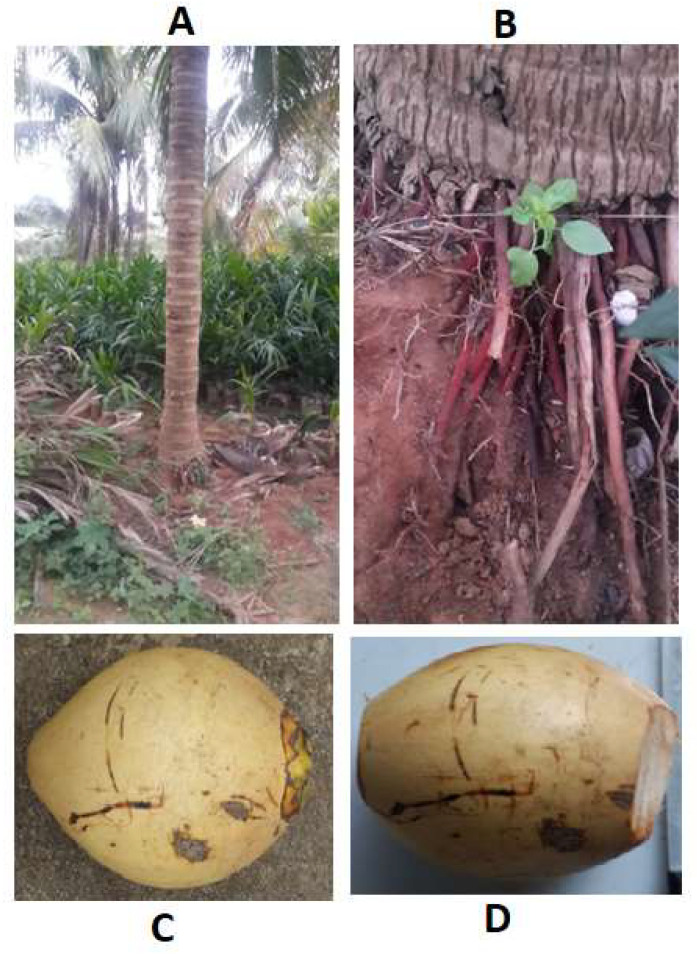
Sources of tissues for RNA segregation. (**A**) Coconut tree; (**B**) coconut root, from which samples were taken and preserved in liquid nitrogen for RNA extraction; (**C**) mature coconut; and (**D**) samples of coconut mesocarp were taken and preserved in liquid nitrogen for RNA extraction.

The use of dichloromethane in the extraction buffer successfully isolated RNA with exceptional purity from the roots of mature coconut tree (Figure 1A,B) and mesocarp (Figure 1C,D) in significant quantities. Additionally, the method effectively isolated RNA from coconut endosperm, leaves, apples, and buds. Table 1 indicates that higher RNA amounts were extracted using the TRIZOL method from the mature coconut mesocarp (346.7 ± 43.18 µg/g FW) and roots of mature coconut tree (140.3 ± 6.79 µg/g FW), but the quality, as reflected by A260/280 ratio (1.26 ± 0.166 and 1.42 ± 0.015), was quite low.

Similarly, the A260/280 ratios of the RNA extracted from the mature coconut tree roots and mature coconut mesocarp using RNA plant (0.76 ± 0.165 and 1.02 ± 0.002) and CTAB (0.83 ± 0.052 and 0.92 ± 0.094) were also very low. Moreover, the quantities of extracted RNA from the mentioned tissues using the RNA plant (5.1 ± 1.86 µg/g FW and 47.8 ± 15.78 µg/g FW) and CTAB (18.9 ± 1.15 µg/g FW and 11.4 ± 1.33 µg/g FW) were minimal.

In contrast, the dichloromethane method successfully isolated RNA from mature tree roots (120.7 ± 3.18 µg/g FW) and the mature mesocarp of coconut (134.6 ± 0.61 µg/g FW) in substantial quantities. The purities of RNA by the dichloromethane method from mature tree roots (1.95 ± 0.012) and mature mesocarp (1.98 ± 0.028) were also high (Table 1). Furthermore, the dichloromethane method was successfully extended to other coconut tissues, which exhibited similar results (Table 2). The concentration of RNA from coconut endosperm was 120.4 ± 2.30 µg/g FW (A260/280 ratio = 2.01 ± 0.060); coconut leaf was 172.9 ± 0.95 µg/g FW (A260/280 ratio = 1.97 ± 0.017); coconut apple was 298.2 ± 2.83 µg/g FW (A260/280 ratio = 1.91 ± 0.050); and coconut bud was 175.7±1.64 µg/g FW (A260/280 ratio = 2.05 ± 0.033).

**Figure 2:**
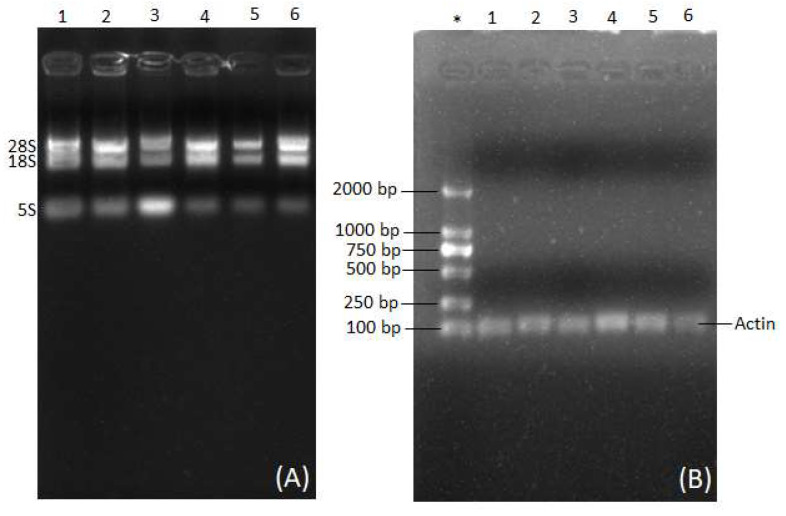
Segregation of RNA and RT-PCR product of isolated RNA from the coconut tissues by dichloromethane method. In (**A**), lane ‘1’ represents roots sample from mature coconut tree; lane ‘2’ represents mesocarp sample from mature coconut; lane ‘3’ represents endosperm sample from mature coconut; lane ‘4’ represents leaf sample of coconut plant; lane ‘5’ represents sample of coconut apple; lane ‘6’ represents sample of coconut bud; and lane ‘*’ in (**B**) represents marker/DNA ladder. (**A**) represents RNA bands that have been obtained after running 3 µL of extracted RNA from various coconut tissues by dichloromethane method on the agrose gel for 12 min; and (**B**) represents the RT-PCR products of RNA isolated from the various coconut tissues by dichloromethane method. Each lane of the agrose gel was loaded with 3 µL of DNA marker or RT-PCR product. The gel was run for 12 min, and the bands were observed in the Gel Doc system.

The electrophoretogram results showed the existence of three RNA bands, namely, 28S, 18S, and 5S rRNA, in the root samples from mature coconut tree and mesocarp samples from mature coconut after extraction with dichloromethane (Figure 1A). Similarly, three RNA bands were observed for samples extracted with dichloromethane from root of mature coconut tree, mesocarp, and endosperm of mature coconut. Likewise, three RNA bands were observed for samples extracted with dichloromethane from coconut leaves, apple, and bud (Figure 2A). 

**Figure 3:**
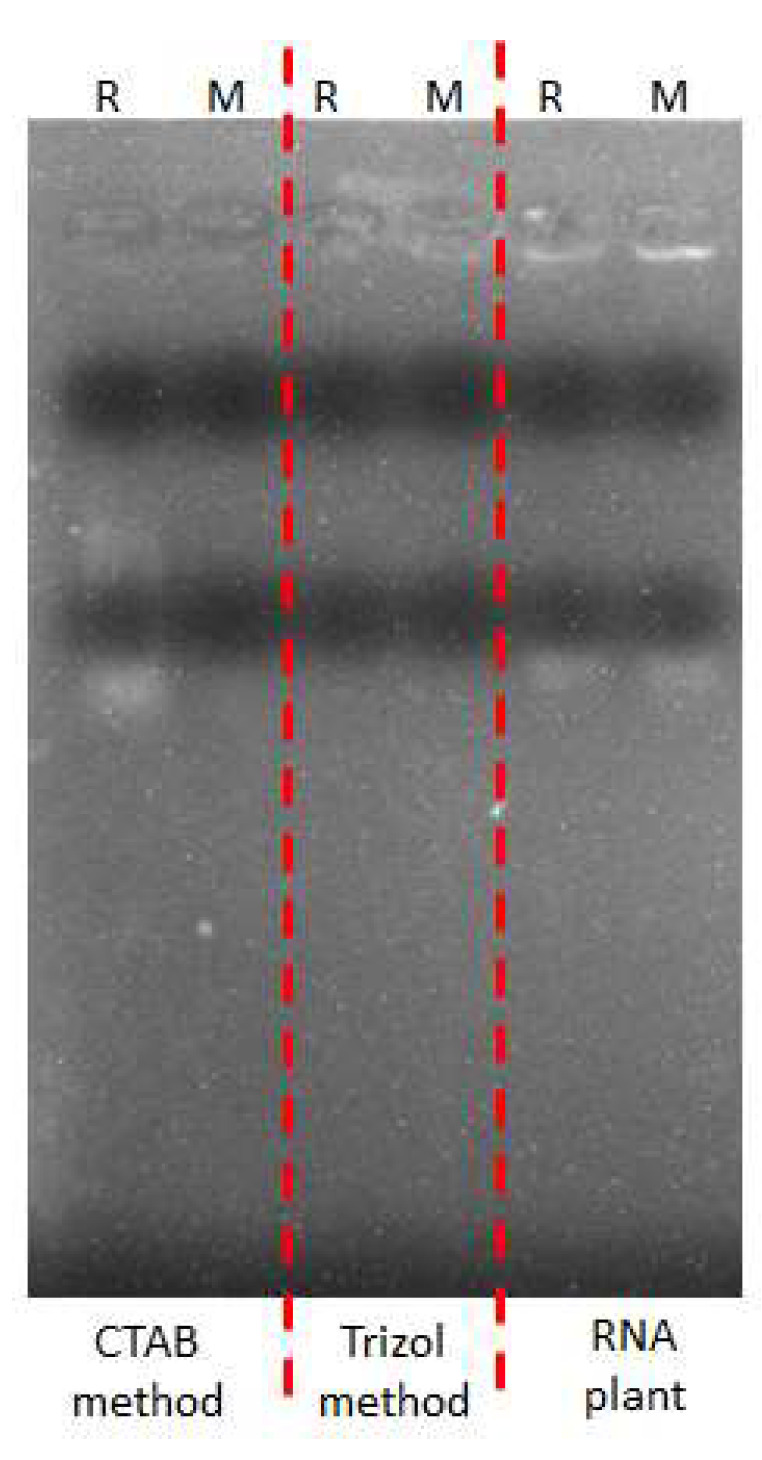
Segregation of RNA from roots of coconut tree and mesocarp of coconut by RNA plant, TRIZOL, and CTAB methods. ‘R’ represents root samples from coconut tree; and ‘M’ represents mesocarp samples from mature coconut. Figure 3 represents RNA bands that have been obtained after running 3 µL of extracted RNA from the roots of coconut tree and mesocarp of coconut by RNA plant, TRIZOL, and CTAB methods on the agrose gel for 12 min.

To confirm the purity of RNA extracted with dichloromethane from the root of mature coconut tree and mesocarp of mature coconut, RT-PCR was performed to synthesize and amplify cDNA. The RNA extracted through the dichloromethane method successfully produced amplified RT-PCR products (Figure 2B). Conversely, the RNA plant kit, TRIZOL, and CTAB failed to produce quality RNA, as shown in Figure 3. 

## 5. Discussion

For RNA sequencing or constructing a cDNA library, it is crucial to have pure RNA. Additionally, when profiling gene expression, the significance of pure RNA cannot be overlooked [18,19,20]. Over time, various methods have been developed and patented to obtain pure RNA from complex tissues of animals and plant species. Additionally, extracting RNA with exceptional purity in substantial amounts from the young tissues of plant species from the Palmacea family presents a considerable challenge. Given the diversity of germplasm in Wenchang, a tropical region in China known for its elite class of coconuts, exploring the molecular traits of the fruit is particularly interesting. As mentioned earlier, studying plants at the molecular level necessitates pure RNA for follow-up studies. The biotechnology team at the Chinese Academy of Tropical Agriculture Sciences has achieved successful extraction of pure RNA of the intricate coconut tissues, including coconut leaves, endosperm, apple, and buds [7,10,13]. Recently, our lab attempted to retrieve superior RNA from the roots of mature coconut trees, as well as from the mesocarp and endosperm of mature coconuts, using various well-established methods, but, unfortunately, these attempts were unsuccessful. In response to the challenge of recovering RNA from the lignified matrices of the coconut, we devised a novel approach based on dichloromethane extraction. This method has proven to be cost-effective, robust, and time-saving, enabling the retrieval of premium RNA from the lignified roots and mesocarp of coconut. 

The presence of both macro- and micro-biomolecules in varying amounts within plant species underscores the need for the appropriate method to isolate pure RNA. In fact, the extraction of pure RNA faces obstacles from RNA-binding proteins impeding complete RNA retrieval [21], ribonucleases leading to RNA degradation [22], and protein contamination disrupting concentration assessment and subsequent uses [23]. In addition, samples loaded in lipids can hamper cell lysis and RNA liberation into the extraction buffer [24]. To offset this, detergents or chaotropic agents should be exploited to disband lipid membranes [25]. Lipids with polyunsaturated fatty acids are susceptible to oxidation, yielding products that can disintegrate RNA during isolation, possibly cutting its quality [26]. Moreover, carbohydrates can augment sample viscosity and stickiness, obscuring cell disturbance [27]. This may hinder proficient cell lysis and RNA discharge during extraction [28]. Carbohydrates can also associate with RNA in precipitation, provoking adulteration and impacting subsequent uses [29]. Carbohydrate associations and extraction agents might obstruct RNA precipitation and purification [28]. Also, the existence of polyphenols might disrupt spectrophotometric-measuring methods for RNA, owning to their absorbance attributes [30]. In practice, the productivity of RNA segregation protocols can significantly vary between different species and tissues. Furthermore, the presence of macro- and micro-biomolecules not only hinders RNA segregation but can also lead to RNA degradation. Therefore, the crucial factor lies in the proper composition of the extraction buffer, including chaotropic agents, polysaccharides, lipids, and polyphenol-binding agents. As is widely recognized, woody tissues are highly lignified, which could potentially be one of the reasons affecting the capability of the RNA extraction column used in the RNA plant (a commercial kit). In the same manner, the CTAB buffer mixture [2] and Trizol could be lacking necessary elements in the formulation that are crucial for extracting RNA from the roots of mature coconut trees and the mesocarp of mature coconut fruits. On the contrary, the RNA extraction buffer, which contained dichloromethane, encompassed all the essential elements for efficiently removing macro- and micro-molecules. Indeed, the presence of dichloromethane in the buffer skillfully retrieved RNA of the woody tissues of the coconut tree roots and the mesocarp of the coconut fruits, which are rich in secondary cell walls. The technique has been proven for RNA isolation from various coconut tissues, including roots, mesocarp, and endosperm of mature coconut fruits, as well as coconut leaves, apples, and buds. Moreover, it has the potential to be extended for the extraction of RNA from other plant and animal species as well.

## 6. Conclusions

In conclusion, the dichloromethane-based method demonstrated its effectiveness in isolating high-quality RNA from mature coconut tree roots and mesocarp, as well as from various other coconut tissues. This method proved to be a valuable alternative to other conventional extraction protocols that showed low yields and poor RNA quality from these lignified tissues. Furthermore, the success of this method opens up possibilities for its application in extracting pure RNA from other plant and animal species, providing a valuable tool for molecular studies and gene expression profiling.

## Figures and Tables

**Table 1:** Concentration and quality of RNA from mature coconut tree root and mature coconut mesocarp by various methods.

Method	Sample Tissue	Conc. (µg/g FW)	A260/280 Ratio
**Dichloromethane**	Mature coconut tree roots	120.7 ± 3.18	1.95 ± 0.012
	Mature coconut mesocarp	134.6 ± 0.61	1.98 ± 0.028
**RNA plant**	Mature coconut tree roots	5.1 ± 1.86	0.76 ± 0.165
	Mature coconut mesocarp	47.8 ± 15.78	1.02 ± 0.002
**TRIZOL**	Mature coconut tree roots	140.3 ± 6.79	1.42 ± 0.015
	Mature coconut mesocarp	346.7 ± 43.18	1.26 ± 0.166
**CTAB**	Mature coconut tree roots	18.9 ± 1.15	0.83 ± 0.052
	Mature coconut mesocarp	11.4 ± 1.33	0.92 ± 0.094

A 1 μL drop was loaded onto the NanoDrop 2000. Each data point represents the mean of triplicated data with ± standard deviation.

**Table 2:** Concentration and quality of RNA from coconut endosperm, leaf, apple and bud by the dichloromethane method.

Sample	Conc. (µg/g FW)	A260/280 Ratio
Coconut endosperm	120.4 ± 2.30	2.01 ± 0.060
Coconut leaf	172.9 ± 0.95	1.97 ± 0.017
Coconut apple	298.2 ± 2.83	1.91 ± 0.050
Coconut leaf bud	175.7 ± 1.64	2.05 ± 0.033

A 1 µL drop was loaded onto the NanoDrop 2000. Each data point represents the mean of triplicated data with ± standard deviation.

## Data Availability

All the data are included in the manuscript.
